# Effect of intra-operative Magnesium sulphate on the occurrence of post-operative delirium and insomnia in patients undergoing lumbar fixation: a randomized controlled trial

**DOI:** 10.1186/s12871-024-02579-6

**Published:** 2024-08-13

**Authors:** Wael Fathy, Mona Hussein, Rehab Elanwar, Hatem Elmoutaz, Donya A Abdelsadek, Dina Y Kassim

**Affiliations:** 1 Department of Anesthesiology, Surgical ICU and Pain Management, Beni-Suef University, Salah Salem Street, Beni-Suef, 62511 Egypt; 2 Department of Neurology, Beni-Suef University, Beni-Suef, Egypt; 3 Neuro Diagnostic Research Center, Beni-Suef University, Beni-Suef, Egypt

**Keywords:** Mg sulphate, Postoperative delirium, Postoperative insomnia, MDAS, QEEG

## Abstract

**Background:**

Over the last two decades, a large body of literature has focused on studying the prevalence and outcome of the postoperative delirium and sleep disturbance. The aim of this work was to evaluate the effect of intraoperative administration of Magnesium sulphate on the occurrence of post-operative delirium and insomnia in patients undergoing lumbar fixation.

**Methods:**

This prospective randomized controlled trial was carried out on 80 patients indicated for lumbar fixation; 40 of them received conventional general anesthesia with extra administration of intraoperative magnesium sulphate (Mg sulphate group), and the other 40 received conventional general anesthesia only (control group). Both groups were submitted to pre-operative assessment of depression using Beck Depression inventory (BDI) scale, pre-operative assessment of fatigue using a fatigue questionnaire, pre- and post-operative assessment of insomnia using Insomnia severity index (ISI), post-operative assessment of delirium using Memorial delirium assessment scale (MDAS), post-operative assessment of pain using Visual Analogue Scale (VAS), and pre- and post-operative Quantitative electroencephalography (QEEG).

**Results:**

Mg sulphate administration, age, pre-operative BDI, pre-operative ISI, and post-operative VAS were independent predictors of post-operative ISI (P-value < 0.001, 0.047, 0.021, < 0.001, and < 0.001 respectively). Age and post-operative VAS were independent predictors of post-operative MDAS (P-value = 0.008, 0.013 respectively). Mg sulphate administration and pre-operative ISI were independent predictors of post-operative VAS (P-value = 0.010, 0.006 respectively).

**Conclusion:**

There was a significant relationship between intraoperative Mg sulphate administration and both post-operative insomnia and pain in unadjusted and adjusted analysis.

**Supplementary Information:**

The online version contains supplementary material available at 10.1186/s12871-024-02579-6.

## Introduction

Post-operative delirium and insomnia are well-known complications in patients undergoing either major or minor surgical. Over the last two decades, a large body of literature has focused on studying their prevalence and outcome [1]. The frequency of post-operative delirium ranged from 10 to 50%. It is defined as delirium occurring 24 to 72 h after surgery [2]. There are multiple risk factors for developing postoperative delirium including pre-existing dementia, old age, medical co-morbidities, and psycopathological symptoms. The diagnosis and treatment of post-operative delirium is critically important because it was reported to be associated with post-operative cognitive decline, insomnia, prolonged hospital stay, and increased mortality (11% increasing in the risk of death at 3 months and up to a 17% increased risk of death at 1 year) [3]. 

Post-operative sleep disturbances are featured by insomnia‚ hypersomnia‚ narcolepsy‚ and changed sleep structure [4, 5]. There are a lot of risk factors associated with post-operative sleep disturbance such as patient age, preoperative comorbidity, severity of surgical trauma, postoperative pain, postoperative complications and presence of pre-operative fatigue and depression [6]. Insomnia is one of the most prevalent health problems during pre-operative period and after post-operative recovery. It can lead to an increased incidence of post-operative complications such as delayed recovery, anxiety, and delirium [7].

Animal studies revealed that Magnesium can regulate melatonin production which is a key player in sleep wake cycle [8]. Magnesium is known to be an essential cofactor for many enzymatic reactions‚ especially those that are involved in neurotransmitter synthesis and energy metabolism [9, 10]. 

Magnesium supplements were used to improve delirium and insomnia among elderly [11]. Low dietary Magnesium intake was reported to be significantly associated with depression which is a well-known risk factor for insomnia [12]. Also, using Magnesium sulphate as an adjuvant was significantly associated with fewer analgesic requirements and reduction of postoperative pain which can improve quality of sleep [13]. 

The aim of this work was to evaluate the effect of intraoperative administration of Magnesium sulphate on the occurrence of post-operative delirium and insomnia in patients undergoing lumbar fixation under general anesthesia. The second objective was to identify the potential predictors of postoperative delirium and insomnia in those patients.

## Methods

### Study design

This prospective randomized controlled trial was conducted on 80 patients indicated for lumbar fixation. The selected patients were randomly assigned to one of the following two groups; the first group received conventional general anesthesia with extra administration of intraoperative magnesium sulphate (40 participants) (Mg sulphate group), and the second group received conventional general anesthesia only (40 participants) (control group). Randomization in this study was done using a closed opaque envelope technique. The patients were recruited from the orthopedic surgery department, Beni-Suef University Hospital, in the period from December 2022 to March 2023. The study was registered in ClinicalTrials.gov on 30/11/2022; this is the identification number NCT05632159.

### Eligibility criteria

This study was carried out on 80 ASA I-II patients indicated for lumbar fixation. The following patients were excluded from the study: patients having major intraoperative ventilatory or hemodynamic fluctuations, patients who developed major postoperative bleeding or hypovolemic shock, patients with sleep apnea syndrome, patient with a history of concomitant medical or metabolic illness known to affect sleep (e.g., thyroid, chest or cardiac disorders), patients with a history of neurodegenerative disease or psychiatric disorders, patients with a current history of illicit drug use, patients using hypnotic, anxiolytic, or antipsychotic drugs, and patients having allergy to any of the used drugs in the study. Pregnant patients were also excluded.

### Clinical assessment

1. **Assessment of depression using Beck Depression inventory (BDI) scale** [14]: It was done for all included patients one day before surgery by a psychiatrist who was blinded to the type of intervention. BDI consists of 21 questions about the individual`s mood in the last week. Each question has 4 possible answer choices, ranging in intensity from 0 to 3.
2. **Assessment of fatigue using a fatigue questionnaire** [15]: It was done for all included patients one day before surgery by a neurologist who was blinded to the type of intervention. It consists of 14-item that assess both physical and mental fatigue. Physical fatigue represents items 1–7, whereas mental fatigue represents items 8–11. Scores for physical and mental fatigue are the sum of the representative items for each section. The total score is the sum all items.
3. **Assessment of insomnia using Insomnia severity index (ISI)** [16]: It was done for all included patients one day before and two weeks after surgery by a neurologist who was blinded to the type of intervention. ISI is a 7-item scale measuring insomnia severity over the previous 2 weeks. Each item is rated on a 5-point scale ranges from 0 (no problem) to 4 (very severe problem). The maximum possible score is 28.
4. **Assessment of postoperative delirium using Memorial delirium assessment scale (MDAS)** [17]: It was done for all included patients between 24 h and 48 h after surgery by a neurologist who was blinded to the type of intervention. MDAS is a 10-item scale that is designed to assess the severity of delirium. Each item is rated on a 4-point scale ranges from 0 (no) to 3 (severe impairment). The maximum possible score is 30.
5. **Assessment of pain using Visual Analogue Scale (VAS)** [18]: It was done for all included patients 24 h after surgery by a neurologist who was blinded to the type of intervention. It is a 10 centimeters horizontal line used for evaluating pain intensity. It has two endpoints: ‘no pain’ and ‘the worst experienced pain’. The patient was asked to mark his pain severity on this line between the two endpoints.

### Neurophysiological assessment

Electroencephalography (EEG) and Quantitative electroencephalography (QEEG) were done for all included patients before, and between 24 h and 48 h after surgery. EEG and QEEG were carried out in the Neuro Diagnostic & Research Center (NDRC), Beni-Suef University Hospital, using Nihon Kohden EEG Japan machine combined with Neuroguide QEEG software. EEG recording was carried out for 20 min in a quiet room while the subject was relaxed with their eyes closed for 5 min and opened for 5 min to insure a high arousal level. The EEG scalp electrodes were applied according to the international 10–20 system using an ear lobe electrode as a reference. The absolute and relative powers of 9 electrodes (F7, F8, T3, T4, O1, O2, Fz, Cz, Pz) were studied in the following frequency bands: delta (0.5–3 Hz), theta (4–7 Hz) and alpha (8–12 Hz) bands.

### Anesthesia technique

A routine preoperative laboratory work up and electrocardiograms were done to the selected patients. In the operating theatre, intravenous cannula was inserted and intravenous fluids were given. Each selected patient was connected to a monitor where the following preoperative readings were taken: HR, NIBP, and SpO2. The patients were randomly recruited into one of the following two groups:

### Group 1

- Induction of anesthesia was done by injection of fentanyl 2 µg/kg, propofol1.5-2.5 mg/kg and atracurium 0.5 mg/kg. The selected patient was intubated with cuffed oral endotracheal tube. A loading dose of intraoperative Magnesium sulphate 30 mg /kg was administered over 10 min followed by as a maintenance dose of 10 mg /kg/h [19–23]. Careful monitoring of those patients was done to detect any clinical signs of magnesium toxicity such as intra-operative bradycardia or hypotension or post-operative nausea, vomiting, disappearance of deep tendon reflexes, hypotension, ECG changes, or respiratory depression.
- Sevoflorane1.5–2% in mixture of O2 and Air (70: 30) was used as a maintenance of anesthesia.

### Group 2

- Induction of anesthesia was done by injection of fentanyl 2 µg/kg, propofol1.5-2.5 mg/kg and atracurium 0.5 mg/kg. The selected patient was intubated with cuffed oral endotracheal tube.
- Sevoflorane1.5–2% in mixture of O2 and Air (70: 30) was used as a maintenance of anesthesia.

Volume controlled ventilation in both Mg and control groups was done to maintain an end tidal CO2 of 35–45 mmHg. IV neostigmine 0.04 mg/kg and atropine 0.02 mg/kg were used to reverse neuromuscular blockade at the end of surgery. Extubation was done when the patient was fully awake then the patient was transferred to post anesthesia care unit (PACU) where they connected to a nasal canula to receive oxygen 4–5 L/min. Postoperatively, all the included patients received intramuscular diclofenac potassium 75 mg every 12 h and paracetamol infusion 1 gm every eight hours.

### Outcomes of the study

The primary outcome of this study was to evaluate the effect of intraoperative administration of Magnesium sulphate on the occurrence of post-operative delirium and insomnia in patients undergoing lumbar fixation under general anesthesia.

The secondary outcome was to identify the potential predictors of postoperative delirium and insomnia in those patients.

### Sample size calculation

The sample size calculation for this trial was done using G*Power version 3.1.9.2 Software. The probability of type I error (α) was 5%, effect size d = 0.742, noncentrality parameter λ = 3.319, Critical t = 1.665, and Df = 78. A total sample size of 40 for each group was required to obtain a statistical power of the study (1–β) 95%.

### Statistical analysis

The data were be coded and entered using SPSS version 22. Descriptive data were presented as mean ± SD for quantitative variables and number (%) for categorical variables. Independent sample t- test was used for comparison between Mg sulphate and control groups in quantitative variables. The P-values in Table (2) were adjusted for multiple testing by performing the Benjamini-Hochberg procedure. Paired sample t- test was used to compare paired quantitative variables. Chi square test was used to compare between Mg sulphate and control groups in categorical variables. Mixed ANOVA test was used for comparing paired data in Mg sulphate and control groups. Multiple linear regression model was used to identify the predictors of post-operative ISI, post-operative MDAS, and post-operative VAS in all included patients in both Mg sulphate and control groups. P value ≤ 0.05 is considered statistically significant.

## Results

### Demographics, pre- and intra-operative data of the included patients in both mg sulphate and control groups

This prospective randomized controlled trial was conducted on 80 patients indicated for lumbar fixation; 40 of them received conventional general anesthesia with extra administration of intraoperative Mg sulphate (Mg sulphate group), and the other 40 received conventional general anesthesia only (control group). There were no statistically significant differences between both groups regarding age (P-value = 0.167), sex (P-value = 0.813), BMI (P-Value = 0.220), preoperative BDI (P-value = 0.558), preoperative fatigue (P-value = 0.206), preoperative ISI ( P-value = 0.084), the duration of the operation (P-value = 0.606), intraoperative MAP (P-value = 0.302), or intraoperative HR (P-value = 0.127) (Table 1).

**Table 1:** Demographics, pre- and intra-operative data of the included patients in both Mg sulphate and control groups

		Mg sulphate group (*n* = 40)	Control group (*n* = 40)	*P*-value
Age [mean (SD)]		46.11 (11.89)	42.20 (10.41)	0.167
Sex	Males [n (%)]	27 (67.5%)	26 (65.0%)	0.813
	Females [n (%)]	13 (32.5%)	14 (35.0%)	
BMI [mean (SD)]		29.21 (3.54)	28.15(3.34)	0.220
Preoperative BDI [mean (SD)]		18.45 (6.55)	21.06 (7.28)	0.134
Preoperative fatigue [mean (SD)]		19.25 (6.02)	21.03(5.02)	0.206
Preoperative ISI [mean (SD)]		15.42 (4.42)	17.33(4.26)	0.084
Duration of the operation [mean (SD)]		107.14(30.63)	111.00 (28.95)	0.606
Intra-operative MAP [mean (SD)]		76.37(12.83)	79.26(10.00)	0.302
Intra-operative HR [mean (SD)]		79.74(12.62)	83.83(7.69)	0.127

BDI: Beck’s Depression Inventory, HR: Heart rate, ISI: Insomnia Severity Index, MAP: Mean arterial blood pressure. P-value > 0.05 (non-significant)

### Post-operative clinical assessment of the included patients in both mg sulphate and control groups

In unadjusted analysis, Mg sulphate group had significantly lower post-operative ISI score, post-operative MDAS score, and post-operative VAS score in comparison to control group (P-value < 0.001, 0.026, 0.001 respectively) (Table 2).

**Table 2:** Post-operative clinical assessment of the included patients in both Mg sulphate and control groups

	Mg sulphate group (*n* = 40)	Control group (*n* = 40)	*P*-value
Post-operative ISI [mean (SD)]	17.45 (4.32)	22.40(3.29)	< 0.001*
Post-operative MDAS [mean (SD)]	8.17 (3.02)	10.03(3.53)	0.026*
Post-operative VAS [mean (SD)]	5.17(2.09)	6.93(1.83)	0.001*

ISI: Insomnia Severity Index, MDAS: Memorial Delirium Assessment Scale, VAS: Visual analogue scale. *P-value ≤ 0.05 (significant). The P values were adjusted for multiple testing using Benjamini and Hochberg procedure

In both Mg sulphate group and control group, there was a significant post-operative increase in the score of the ISI (P-value < 0.001, < 0.001), but the post-operative increase was significantly lower in Mg sulphate group in comparison to control group (P-value = 0.001) (Table 3).

**Table 3:** Pre and post-operative ISI in both Mg sulphate and control groups

	Preoperative ISI [mean (SD)]	Postoperative ISI [mean (SD)]	*P*- value	*P*- value between groups
Mg sulphate group	15.42 (4.42)	17.45 (4.32)	< 0.001*	0.001
Control group	17.33(4.26)	22.40 (3.29)	< 0.001*	

ISI: Insomnia Severity Index *P-value ≤ 0.05 (significant)

### Pre- and post-operative quantitative EEG parameters in both mg sulphate and control groups

There were no statistically significant differences between Mg sulphate group and the control group regarding the post-operative change in quantitative EEG parameters except in A Abs T4 (P-Value = 0.047). There were also statistically significant differences between pre and post operative A Abs O1 and A Abs O2 in Mg group (P-Value = 0.048,0.030 respectively) (Supplement 1).

### Predictors of post-operative ISI, post-operative MDAS, and post-operative VAS in all included patients in both mg sulphate and control groups

Multiple linear regression model was used to identify the predictors of post-operative ISI, post-operative MDAS, and post-operative VAS in all included patients in both Mg sulphate and control groups. Mg sulphate administration, age, BMI, duration of the operation, pre-operative BDI, fatigue, and ISI, and post-operative VAS were used as the independent variables.

Mg sulphate administration, age, pre-operative BDI, pre-operative ISI, and post-operative VAS were independent predictors of post-operative ISI (P-value < 0.001, 0.047, 0.021, < 0.001, and < 0.001 respectively) (Table 4).

**Table 4:** Predictors of post-operative ISI, post-operative MDAS, and post-operative VAS in all included patients in both Mg sulphate and control groups

Dependent variables	Independent variables	B	P-value	95%CI		VIF
				Lower bound	Upper bound	
Post-operative ISI	(constant)	9.296	< 0.001	5.119	13.474	
	Mg sulphate	-2.184	< 0.001*	-3.303	-1.065	1.435
	Age	-0.059	0.047*	-0.117	-0.001	1.980
	BMI	0.000	0.995	-0.157	0.156	1.336
	Duration of the operation	0.005	0.616	-0.014	0.023	1.343
	Pre-operative BDI	-0.101	0.021*	-0.186	-0.016	1.605
	Pre-operative Fatigue	0.006	0.924	-0.115	0.127	2.104
	Pre-operative ISI	0.634	< 0.001*	0.484	0.784	2.007
	Post-operative VAS	0.883	< 0.001*	0.598	1.167	1.708
Post-operative MDAS	(constant)	-2.981	0.228	-7.883	1.920	
	Mg sulphate	-0.850	0.200	-2.163	0.463	1.435
	Age	0.095	0.008*	0.026	0.163	1.980
	BMI	-0.007	0.943	-0.190	0.177	1.336
	Duration of the operation	0.008	0.484	-0.014	0.029	1.343
	Pre-operative BDI	0.087	0.088	-0.013	0.187	1.605
	Pre-operative Fatigue	0.050	0.485	-0.092	0.192	2.104
	Pre-operative ISI	0.145	0.104	-0.031	0.322	2.007
	Post-operative VAS	0.428	0.013*	0.094	0.762	1.708
Post-operative VAS	(constant)	3.327	0.084	-0.464	7.118	
	Mg sulphate	-1.315	0.010*	-2.298	-0.333	1.275
	Age	0.006	0.819	-0.048	0.061	1.978
	BMI	-0.012	0.866	-0.158	0.133	1.336
	Duration of the operation	-0.011	0.183	-0.028	0.005	1.302
	Pre-operative BDI	0.001	0.974	-0.078	0.081	1.605
	Pre-operative Fatigue	0.079	0.159	-0.032	0.190	2.032
	Pre-operative ISI	0.188	0.006*	0.057	0.319	1.752

BMI: body mass index, BDI: Beck’s Depression Inventory, ISI: Insomnia Severity Index, MDAS: Memorial Delirium Assessment Scale, VAS: Visual analogue scale. *P-value ≤ 0.05 (significant)

Age and post-operative VAS were independent predictors of post-operative MDAS (P-value = 0.008, 0.013 respectively) (Table 4).

Mg sulphate administration and pre-operative ISI were independent predictors of post-operative VAS (P-value = 0.010, 0.006 respectively) (Table 4).

Magnesium sulphate group had lower post operative ISI score {mean difference − 2.2 (95% CI -3.3 to -1.1)} and post-operative VAS score {mean difference − 1.3 (95% CI -2.3 to -0.3)}.

However post-operative MDAS scores were similar between Magnesium sulphate groups and control group {mean difference − 0.85 (95% CI -2.2 to 0.5)} (Table 4).

## Discussion

Post-operative delirium and insomnia are increasingly being recognized as one of the post-operative complications that have a potential severe impact on the patients’ post-operative quality of life. They are associated with longer hospital stays, higher healthcare costs, worse functional outcomes, and increased mortality [7]. 

The aim of this work was to evaluate the effect of intraoperative administration of Magnesium sulphate on the occurrence of post-operative delirium and insomnia in patients undergoing lumbar fixation under general anesthesia.

Assessment of post-operative delirium in this study was done by MDAS. While in unadjusted analysis, a significant association (P-value = 0.026, Table 2) between intraoperative Mg sulphate administration and post-operative delirium was seen,, after adjustment for confounders, such association was not seen (P-value = 0.2, Table 4).

Several studies have investigated the efficacy of Mg sulphate in reducing postoperative delirium and agitation, however most of these studies were performed following pediatric anaesthesia.

Similar findings were obtained by Lee et al. who conducted a study about the effect of Mg supplementation on the occurrence of emergence delirium and postoperative pain in children undergoing strabismus surgery. They gave the included patients 30 mg/kg loading dose of Magnesium sulphate over 10 min then 10 mg/kg every one hour. They found that Mg supplementation had no significant impact on the occurrence of delirium or postoperative pain [24]. 

In contrast, Elsonbaty and lsonbaty found that the incidence of postoperative delirium was significantly lower in patients who received intraoperative 30 mg/kg loading dose of Magnesium sulphate in comparison to those who didn’t receive [25].

Also, Abdulatif et al. studied the effect of magnesium sulphate infusion in children undergoing adenotonsillectomy using sevoflurane anesthesia. They found that delirium scores were significantly lower in magnesium sulphate group in comparison to control group [26].

Bondok & Ali, evaluated Mg sulphate in children aged 3–6 years scheduled for elective inguinal hernia repair. No agitation or delirium were reported in Mg sulphate group as compared with control group. The authors concluded that intravenous Mg sulfate infusion significantly reduced the incidence of sevoflurane-induced emergence agitation and delirium [27]. 

The mechanism that can explain why Mg supplementation may decreases the occurrence of postoperative delirium remains to be elucidated. Mg sulphate exerts its sedative and analgesic effects through non-competitive N-methyl-D-aspartate (NMDA) receptor antagonism. It inhibits the entry of calcium and magnesium into cells, thus preventing activation of catabolic enzymes (e.g. endonucleases, phospholipases, proteases), production of free radical and the secondary cascade of excitotoxic neuronal damage [28]. Intravenous Mg sulphate has been found to suppress the increase in brain lactate level and protects the brain from deleterious effects of prolonged hypotension [29] Mg sulphate was also reported to exert its neuroprotective effect through decreasing vascular instability and downregulating inflammatory cascade via reducing the levels of proinflammatory cytokines (TNF-α, IL-6) [30]. 

Assessment of insomnia in this study was done using ISI. In both Mg sulphate and control groups, there was a significant post-operative increase in the score of ISI, but the post-operative increase was significantly lower in Mg sulphate group in comparison to control group (P-Value = 0.001). A significant association was seen between intraoperative Mg sulphate administration and post-operative insomnia in both unadjusted and adjusted analysis (P-value < 0.001, Table 2; P-value < 0.001, Table 4).

Regarding the effect of general anesthesia on sleep, similar findings were obtained by Steinmetz [31]. Exposure of infants to remifentanil and propofol anesthesia was found to impair postoperative sleep quality. Also, exposure to isoflurane, sevoflurane, and halothane, was reported to cause sleep fragmentation [32]. 

Regarding the effect of Mg sulphate on sleep quality, Bhatia et al. found that patients who were administered 50 mg/kg magnesium sulfate during open cholecystectomy had significantly less insomnia during the first postoperative night in comparison to control group [33]. 

Abbasi et al. observed that supplementation of Mg appears to improve insomnia in elderly. They concluded that Mg can be used as an effective therapeutic supplement for treating some sleep disorders in the elderly people [11]. 

In contrast to our findings, Kayalha et al. reported in their study about the effect of IV Mg sulphate on decreasing post-operative opioid requirement, that score of sleep quality was not significant between two groups at the first night after surgery [34]. 

Since postoperative pain is one of the major causes of emergence agitation and delirium. Therefore, eliminating pain may reduce the incidence of Emergence agitation and delirium [35]. 

Assessment of post-operative pain in this study was done using VAS. A significant association was seen between intraoperative Mg sulphate administration and post-operative VAS in both unadjusted and adjusted analysis (P-value = 0.001, Table 2; P-value = 0.010, Table 4). This adjuvant analgesic effect of Mg sulphate might explain its effect in reducing insomnia and delirium postoperatively.

Several studies evaluated the analgesic effect of Mg sulphate. Ghezel-Ahmadi et al. used a loading dose of Mg sulphate (40 mg/kg over 10 min) followed by an infusion over 24 h (10 mg/kg/h) to assess the effect of Mg sulphate on post-thoracotomy pain. Patients who received intraoperative Mg sulphate had less postoperative pain and less opioid consumption compared to patients who did not [36]. Also, Tsaousi et al. observed that postoperative VAS scores were considerably lower in Mg sulphate group in comparison to control group [37]. 

In the present study, Mg sulphate administration, age, pre-operative BDI, pre-operative ISI, and post-operative VAS were found to be independent predictors of post-operative ISI (P-value < 0.001, 0.047, 0.021, < 0.001, and < 0.001 respectively). Age and post-operative VAS were found to be independent predictors of post-operative MDAS (P-value = 0.008, 0.013 respectively). Mg sulphate administration and pre-operative ISI were found to be independent predictors of post-operative VAS (P-value = 0.010, 0.006 respectively).

Cho et al. studied the relation between sleeping disorders and postoperative delirium. The authors found that patients with sleeping disorders have odds ratio of 5.78 of experiencing delirium compared with those who do not [38]. 

Also, the systematic review and meta-analysis performed by Lu et al., reported a positive role of sleep promotion and circadian intervention in decreasing the occurrence of postoperative delirium [39]. 

Thomas et al., had addressed sharing mechanisms between the pre-exisiting sleep disorders and post-operative delirium. After 24-h sleep deprivation, PET imaging showed decreased cerebral metabolism in the thalamus, posterior parietal cortex, and prefrontal cortex. Those brain areas were reported to be involved in the pathophysiology of delirium [40]. 

As regards the effect of fatigue on sleep and delirium, multiple studies found a strong correlation between fatigue and sleep disorders. Sleep is often recognized as an effective countermeasure against fatigue. It is also assumed that the main cause of fatigue in individuals with insomnia is the lack of restorative sleep [41, 42]. Alapin et al. found that fatigue was significantly correlated with sleep duration, sleep efficiency, time spent awake during the night, anxiety, and depression [43]. 

The relationship between sleep and pain is reciprocal, non-restorative sleep leads to increased sensitivity to pain. There is a well-known increase in proinflammatory mediators in patients with sleep disorders, which may exacerbate pain sensation. Multiple studies have revealed that postoperative pain is the most important factor for development of postoperative sleep disturbances [6]. 

In agreement with our results, among many risk factors for postoperative delirium, age was the most significant predictor [44]. . Similar findings were obtained by Morimoto et al. who found that age was an important risk factor for POD [45]. 

The effect of age on the emergence of postoperative delirium could be attributed to the age-related increase drug sensitivity and toxicity in elderly patients compared to young adults [46]. 

In disagreement with our study, Kazmierski et al. suggested that the presence of depressive symptoms may be associated with increased risk of post-operative delirium [47]. 

This study had some limitations. Firstly, the relatively small sample size. Secondly, magnesium level was not measured in the included patients in Mg sulphate group, so we relied only on careful monitoring of those patients to detect any clinical signs of magnesium toxicity. Thirdly, assessment of pain in the current study using VAS may not be the appropriate tool to assess pain in the early post-operative period because post-operative delirium may impact pain assessment at this time. Fourthly, the study focused only on the assessment of insomnia and postoperative delirium and didn’t assess the other post-operative sleep disorders or the post-operative cognitive dysfunction.

## Conclusion

There was a significant relationship between intraoperative Mg sulphate administration and both post-operative insomnia and pain in unadjusted and adjusted analysis. Age, pre-operative BDI, pre-operative ISI, and post-operative VAS were independent predictors of post-operative ISI. Age and post-operative VAS were independent predictors of post-operative MDAS. Pre-operative ISI was an independent predictor of post-operative VAS.

### Electronic supplementary material

Below is the link to the electronic supplementary material.

Supplementary Material 1

## Data Availability

Authors report that the datasets used and/or analyzed during the current study are available from the corresponding author on reasonable request.

## Declarations

Authors report that the content has not been published or submitted for publication elsewhere.
