# Supplementary material for: Effect of intra-operative Magnesium sulphate on the occurrence of post-operative delirium and insomnia in patients undergoing lumbar fixation: a randomized controlled trial
Source: BMC Anesthesiol. 2024 Aug 13;24:289. doi: 10.1186/s12871-024-02579-6 (PMC11320981; doi:10.1186/s12871-024-02579-6)
Supplement: Supplementary file 1 — Supplementary Material 1 [file 12871_2024_2579_MOESM1_ESM.docx]

Pre and post-operative quantitative EEG parameters in both Mg sulphate and control groups

| Quantitative EEG parameters | | Preoperative assessment  [mean (SD)] | Postoperative assessment  [mean (SD)] | P- value | P- value between groups |
| --- | --- | --- | --- | --- | --- |
| D Abs F7 | Mg sulphate group | 20.79 (12.44) | 21.34(16.38) | 0.866 | 0.488 |
|  | Control group | 18.37 (10.58) | 18.89 (13.66) | 0.870 |  |
| D Abs F8 | Mg sulphate group | 24.08 (17.70) | 24.37 (14.37) | 0.949 | 0.810 |
|  | Control group | 25.16 (16.19) | 21.60 (7.60) | 0.318 |  |
| D Abs T3 | Mg sulphate group | 10.34(7.13) | 14.99 (10.95) | 0.069 | 0.258 |
|  | Control group | 10.48(6.21) | 10.24 (6.07) | 0.850 |  |
| D Abs T4 | Mg sulphate group | 12.34 (8.67) | 19.06 (20.59) | 0.206 | 0.184 |
|  | Control group | 11.35 (5.81) | 12.76 (9.86) | 0.533 |  |
| D Abs O1 | Mg sulphate group | 12.57 (8.81) | 15.59(10.44) | 0.247 | 0.516 |
|  | Control group | 13.58 (6.48) | 19.53 (27.81) | 0.309 |  |
| D Abs O2 | Mg sulphate group | 15.09 (11.69) | 20.71 (16.92) | 0.224 | 0.202 |
|  | Control group | 13.87 (7.04) | 14.86 (8.73) | 0.563 |  |
| D Abs Fz | Mg sulphate Group | 22.91 (22.44) | 24.1610 (18.92) | 0.813 | 0.493 |
|  | Control Group | 35.77 (66.34) | 29.47(47.31) | 0.237 |  |
| D Abs Cz | Mg sulphate Group | 5.76 (3.79) | 5.78 (3.20) | 0.987 | 0.431 |
|  | Control group | 8.47 (15.37) | 6.23 (5.64 ) | 0.509 |  |
| D Abs Pz | Mg sulphate Group | 8.29 (5.89 ) | 12.21 (22.68) | 0.454 | 0.629 |
|  | Control Group | 10.26 (12.70) | 7.34 (5.33) | 0.330 |  |
| T Abs F7 | Mg sulphate Group | 5.86(2.89) | 7.66(5.00) | 0.159 | 0.272 |
|  | Control group | 5.79 (2.81) | 5.94 (2.71 ) | 0.844 |  |
| T Abs F8 | Mg sulphate Group | 7.27 (4.27) | 8.42 (4.63) | 0.347 | 0.253 |
|  | Control Group | 6.63 (1.87) | 7.05 (2.54) | 0.421 |  |
| T Abs T3 | Mg sulphate Group | 4.58 (2.71) | 7.22 (5.95 ) | 0.036* | 0.135 |
|  | Control Group | 4.53 (2.27) | 4.45 (2.32 ) | 0.886 |  |
| T Abs T4 | Mg sulphate Group | 4.74 (2.32 ) | 9.90 (14.11 ) | 0.112 | 0.123 |
|  | Control Group | 4.21 (1.46) | 5.27 (3.28) | 0.111 |  |
| T Abs O1 | Mg sulphate Group | 5.31 (3.17 ) | 10.61(14.60 ) | 0.091 | 0.392 |
|  | Control Group | 5.57 (2.27) | 6.99 (7.63) | 0.425 |  |
| T Abs O2 | Mg sulphate Group | 6.32 (3.97) | 13.97(18.26 ) | 0.063 | 0.071 |
|  | Control Group | 5.83 (2.48) | 6.31 (3.35) | 0.491 |  |
| T Abs Fz | Mg sulphate Group | 6.22 (4.48) | 7.25 (4.83) | 0.457 | 0.936 |
|  | Control Group | 7.05 (6.79 ) | 6.68 (6.08) | 0.584 |  |
| T Abs Cz | Mg sulphate Group | 2.83(1.45) | 3.15(1.55) | 0.447 | 0.412 |
|  | Control Group | 3.05 (3.00) | 2.21 (1.13 ) | 0.248 |  |
| T Abs Pz | Mg sulphate Group | 3.01(2.00 ) | 4.87(6.02) | 0.137 | 0.324 |
|  | Control Group | 3.37(2.84) | 2.81 (1.73) | 0.438 |  |
| A Abs F7 | Mg sulphate Group | 4.77(3.24) | 6.40(5.53) | 0.164 | 0.098 |
|  | Control Group | 4.23(3.32) | 3.77 (1.60) | 0.468 |  |
| A Abs F8 | Mg sulphate Group | 5.14(3.07) | 7.28(6.44) | 0.090 | 0.140 |
|  | Control Group | 4.76(2.73) | 4.62 (2.16) | 0.823 |  |
| A Abs T3 | Mg sulphate Group | 4.64 (3.36) | 7.49 (8.82) | 0.116 | 0.242 |
|  | Control Group | 5.10(6.86) | 3.66 (2.24) | 0.341 |  |
| A Abs T4 | Mg sulphate Group | 4.93(3.50) | 11.56(16.48) | 0.080 | 0.047 |
|  | Control Group | 4.10 (3.21) | 4.38 (2.72) | 0.718 |  |
| A Abs O1 | Mg sulphate Group | 7.60(7.52) | 16.29(20.12) | 0.048* | 0.407 |
|  | Control Group | 9.36 (10.94) | 9.32 (11.14) | 0.991 |  |
| A Abs O2 | Mg sulphate Group | 9.11 (7.34) | 22.39(25.99) | 0.030* | 0.063 |
|  | Control Group | 9.56 (13.51) | 8.15 (7.78) | 0.637 |  |
| A Abs Fz | Mg sulphate Group | 3.95(3.54) | 6.51 (6.17) | 0.059 | 0.054 |
|  | Control Group | 3.52 (2.52) | 3.14 (1.95) | 0.574 |  |
| A Abs Cz | Mg sulphate Group | 2.34(1.89) | 3.00 (2.39) | 0.125 | 0.085 |
|  | Control Group | 2.08(1.44) | 1.60 (1.22) | 0.217 |  |
| A Abs Pz | Mg sulphate Group | 3.59(3.12) | 6.00(6.81) | 0.132 | 0.406 |
|  | Control Group | 4.03(5.07) | 3.61 (5.10) | 0.789 |  |
| D/T ratio F7 | Mg sulphate Group | 3.79(2.33) | 2.93(0.94) | 0.077 | 0.686 |
|  | Control Group | 3.20(1.32) | 3.15 (1.68) | 0.853 |  |
| D/T ratio F8 | Mg sulphate Group | 3.23(1.26) | 3.00(1.05) | 0.517 | 0.245 |
|  | Control Group | 3.77(1.60) | 3.29 (1.57) | 0.199 |  |
| D/T ratio T3 | Mg sulphate Group | 2.29(0.78) | 2.39(1.07) | 0.730 | 0.980 |
|  | Control Group | 2.32(0.72) | 2.34 (0.89) | 0.903 |  |
| D/T RatioT4 | Mg sulphate Group | 2.54 (0.83) | 2.38(0.87) | 0.565 | 0.707 |
|  | Control Group | 2.66(0.92) | 2.43 (0.96) | 0.289 |  |
| D/T ratio O1 | Mg sulphate Group | 2.35(0.84) | 1.99(1.03) | 0.196 | 0.138 |
|  | Control Group | 2.56(1.11) | 2.58 (1.35) | 0.955 |  |
| D/T ratio O2 | Mg sulphate Group | 2.39 (0.71) | 2.29 (1.11) | 0.716 | 0.596 |
|  | Control Group | 2.51(1.09) | 2.47(1.21) | 0.774 |  |
| D/T ratio Fz | Mg sulphate Group | 3.70 (1.53) | 3.53 (1.93) | 0.756 | 0.619 |
|  | Control Group | 4.03(1.61) | 3.63(1.65) | 0.187 |  |
| D/T ratio Cz | Mg sulphate Group | 2.27(1.15) | 2.04 (1.01) | 0.464 | 0.175 |
|  | Control Group | 2.50 (1.02) | 2.59 (1.27) | 0.726 |  |
| D/T ratio Pz | Mg sulphate Group | 3.09 (1.65) | 2.63 (1.32) | 0.368 | 0.391 |
|  | Control Group | 2.66(0.83) | 2.62 (0.96) | 0.896 |  |
| T/A ratio F7 | Mg sulphate Group | 1.46(0.64) | 1.43 (0.57) | 0.852 | 0.135 |
|  | Control Group | 1.72 (0.79) | 1.68 (0.55) | 0.766 |  |
| T/A ratio F8 | Mg sulphate Group | 1.65 (0.89) | 1.47 (0.60) | 0.328 | 0.432 |
|  | Control Group | 1.71 (0.80) | 1.72 (0.66) | 0.951 |  |
| T/A ratio T3 | Mg sulphate Group | 1.22 (0.59) | 1.24 (0.56) | 0.858 | 0.284 |
|  | Control Group | 1.42 (0.71) | 1.40 (0.55) | 0.911 |  |
| T/A Ratio T4 | Mg sulphate Group | 1.25 (0.65) | 1.12 (0.54) | 0.371 | 0.251 |
|  | Control Group | 1.43 (0.88) | 1.37 (0.56) | 0.710 |  |
| T/A ratio O1 | Mg sulphate Group | 1.02 (0.63) | 1.02 (0.66) | 0.980 | 0.525 |
|  | Control Group | 1.14 (0.71) | 1.11 (0.60) | 0.832 |  |
| T/A ratio O2 | Mg sulphate Group | 1.00 (0.65) | 0.91 (0.67) | 0.667 | 0.191 |
|  | Control Group | 1.24 (0.89) | 1.16 (0.66) | 0.643 |  |
| T/A ratio Fz | Mg sulphate Group | 2.11 (1.26) | 1.63 (0.81) | 0.110 | 0.156 |
|  | Control Group | 2.60 (2.228) | 2.59(2.18) | 0.981 |  |
| T/A ratio Cz | Mg sulphate Group | 1.64 (0.82) | 1.41 (0.64) | 0.335 | 0.551 |
|  | Control Group | 1.51(0.77) | 1.75 (0.74) | 0.246 |  |
| Pre T/A ratio Pz | Mg sulphate Group | 1.15 (0.73) | 1.19 (0.68) | 0.864 | 0.348 |
|  | Control Group | 1.32 (0.70) | 1.46 (1.30) | 0.504 |  |

*P-value ≤ 0.05 (significant)
